# A Range-Normalization Model of Context-Dependent Choice: A New Model and Evidence

**DOI:** 10.1371/journal.pcbi.1002607

**Published:** 2012-07-19

**Authors:** Alireza Soltani, Benedetto De Martino, Colin Camerer

**Affiliations:** 1 Howard Hughes Medical Institute and Department of Neurobiology, Stanford University School of Medicine, Stanford, California, United States of America; 2 Division of Biology and Computation and Neural Systems, California Institute of Technology, Pasadena, California, United States of America; 3 Division of Psychology and Language Sciences, University College London, London, United Kingdom; 4 Division of the Humanities and Social Sciences, California Institute of Technology Pasadena, California, United States of America; New York University, United States of America

## Abstract

Most utility theories of choice assume that the introduction of an irrelevant option (called the decoy) to a choice set does not change the preference between existing options. On the contrary, a wealth of behavioral data demonstrates the dependence of preference on the decoy and on the context in which the options are presented. Nevertheless, neural mechanisms underlying context-dependent preference are poorly understood. In order to shed light on these mechanisms, we design and perform a novel experiment to measure within-subject decoy effects. We find within-subject decoy effects similar to what have been shown previously with between-subject designs. More importantly, we find that not only are the decoy effects correlated, pointing to similar underlying mechanisms, but also these effects increase with the distance of the decoy from the original options. To explain these observations, we construct a plausible neuronal model that can account for decoy effects based on the trial-by-trial adjustment of neural representations to the set of available options. This adjustment mechanism, which we call range normalization, occurs when the nervous system is required to represent different stimuli distinguishably, while being limited to using bounded neural activity. The proposed model captures our experimental observations and makes new predictions about the influence of the choice set size on the decoy effects, which are in contrast to previous models of context-dependent choice preference. Critically, unlike previous psychological models, the computational resource required by our range-normalization model does not increase exponentially as the set size increases. Our results show that context-dependent choice behavior, which is commonly perceived as an irrational response to the presence of irrelevant options, could be a natural consequence of the biophysical limits of neural representation in the brain.

## Author Summary

While faced with a decision between two options for which you have no clear preference (say, a small cheap TV and a large expensive TV), you are presented with a new but inferior option (say, a medium expensive TV). The mere presence of the new option, which you would not select anyway, shifts your preference toward the expensive large TV. This simple example shows how the introduction of an irrelevant option, called the “decoy,” to the choice set can change preference between existing options, a phenomenon often called the context-dependent preference reversal. A number of models have been proposed to explain context effects. Despite their success, they are either uninformative about the underlying neural mechanisms or they require comparison of every possible pair of option attributes, a computation that is unlikely to be implemented by the nervous system due to its high computational demand and undesirable outcomes when the choice set size increases. Here we present a novel account of the context-dependent preference based on the adjustment of neural response to the set of available options. Moreover, we show results from a novel behavioral task designed to test contrasting predictions of our model and a classic model of context effects.

## Introduction

At the core of many utility theories used in social and biological sciences lies a central axiom, called independence from irrelevant alternatives (IIA). The IIA axiom states that the relative preference between any pair of options does not depend on what other options might be present [1]–[3]. In decision neuroscience, IIA holds in the appealing model in which separate values are computed for each different option, and values are then compared to make a choice [4], [5]. Nevertheless, a wealth of data has clearly shown that the IIA axiom is often violated behaviorally [6], [7]. For example, it has been shown that adding a third “decoy” option into a choice set often results in a predictable shift in the relative preference between the other two options of an initial pair. A striking example is when the decoy option is dominated by one initial option – i.e., all of the new option's attributes are worse than the existing option attributes – but is not dominated by the other initial option. The decoy is an “irrelevant alternative” because it would never be chosen if it is dominated by another option. Introducing such a decoy results in an increased preference for the initial option that dominates the decoy [6], [8]–[10], a phenomenon called the *attraction effect* or the *asymmetric dominance effect*.

Decoy effects can be considered an error in logical reasoning and there is some evidence that they can be exploited by consumer marketing and political strategies [11]–[13]. Interestingly, these effects are not limited to humans [14]–[16], they increase after lesion of the medial orbitofrontal cortex in macaques [17], and they can be mitigated by improving self-control or increasing blood glucose [18]. Considering that under realistic scenarios, choices are usually made in particular contexts [19], exploring the neural mechanisms underlying context-dependent preference is crucial for better understanding of choice behavior in general [20].

Several explanations have been proposed to account for the preference reversal induced by the type of decoy in a choice set. Most of these models are based on verbally-described heuristics and are not mathematically formalized, which makes them difficult to test or generalize to new experimental paradigms [21], [22]. An exception is the context-dependent “advantage” (CDA) model of Tversky and Simonson that coherently accounts for attraction and other context effects [7]. The CDA relies on the comparison between different attributes of the available options to account for context effects [23]. The CDA model is the precursor of more elaborate connectionist models such as the leaky competing accumulator (LCA) model [24], [25] or the decision field theory (DFT) [26], [27]. All these models aim to account for many types of context effects such as attraction, similarity, and compromise effects within a single framework [28]. The two popular connectionist models, the LCA and DFT, differ in a number of key features, such as the requirement of loss aversion, but like the CDA model, their core mechanism is comparison between each pair of option attributes. In most cases, psychological models such as CDA, LCA, and DFT, successfully reproduce the behavioral observations that they aim to explain. However comparing all attributes between all pairs of options in the choice set is computationally demanding, especially as the number of options and attributes grows. Other models of choice avoid these demands by assuming limited sequential attribute comparison (e.g., elimination-by-aspects [29], for which there is evidence [30]), but those models cannot explain the attraction effect.

We propose a new model to explain context effects, based on known biophysical limits of neural representation. The guiding presumption in our range-normalization (RN) model is that subjective values of option attributes are encoded in the firing rate of neural populations, rather than other aspects of neural firing [31]. If so, mental representations of subjective values will be bound by the same biophysical limits that govern neural representations. Namely, neural responses are bound from below by zero and from above by a few hundred spikes per second and, therefore, neurons can only represent a set of stimuli using a limited range of firing rates. Faced with a new set of stimuli to encode, however, neurons can adjust their *dynamic range* (i.e. interval between threshold and saturation points) to represent these stimuli distinguishably. We propose that this adjustment mechanism, which we call *range normalization*, is the principal neural mechanism underlying context-dependent effects.

Normalization of the neural response is common in vision and other sensory modalities, and could be a more widespread property of neural representations [32]. To account for context effects, the range-normalization mechanism we propose here is computationally easier than comparison of all pairs of option attributes, since only the two most extreme attribute values are needed to compute the range. We implement a specific functional form of range normalization and test predictions of the outcome model using a novel within-subject design.

We first describe experimental results that demonstrate within-subject decoy effects and reveal some new properties of these effects (correlation between effects across types of decoys and decoy distance). Second, we describe the CDA model, how attribute comparison gives rise to context effects in this model, and its predictions in our experimental paradigm. Third, we present our RN model and its predictions for context effects. Finally, we describe new, contrasting predictions of the CDA and RN models about the influence of choice set size on context effects and the neural plausibility of these models.

## Results

### The experimental paradigm to test within-subject decoy effects

Our experimental paradigm consisted of two tasks: an initial estimation task and the decoy task. We used the subject's choice from the estimation task to calculate the subject's attitude toward risk in order to tailor subject-specific target (T) and competitor (C) gambles that are equally preferred (see below). This step is necessary because context effects are most strongly demonstrated when T and C are equally valuable. In the second part of the experiment (decoy task), we assessed the preference between jittered versions of the T and C gambles in the presence of a third decoy gamble (see Methods for more details).

### Behavioral results from the estimation task

During the estimation task, the subject was presented with two options. These options were risky monetary gambles, described by probability *p* of winning a monetary reward of magnitude *M*, denoted 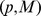. On each trial, the subject selected between pairs of gambles, always consisting of one fixed low-risk gamble, (0.7, $20), and one high-risk gamble, (0.3, $M), for many different values of *M* (see Methods for more details).

The data analysis of the estimation task confirmed that all subjects appeared to understand the task and respond to changes in magnitude, preferring the high-risk gamble when its reward magnitude was large, but not when its reward magnitude was small (Figure S1 in Text S1). Logistic fitting of these choices yielded a subject-specific value of the high-risk gamble magnitude *M* for which the low- and high-risk gambles are equally subjectively valuable. (Figures S2A and S2B in Text S1). Across subjects, we found a wide range of values for the indifference high-risk magnitude and the sensitivity to reward magnitude (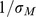), but these two quantities were not significantly correlated (*p = 0.33*) (Figure S2C in Text S1).

As a validity check, we computed the relative expected utility of each pair of gambles (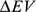), and divided the pairs into sets with 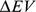 either greater than 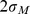 (easy choice pairs), or less than 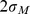 (hard choice pairs). If value is being inferred accurately, response times (RTs) should be slower for hard choice pairs that are close in subjective value. As predicted, the average RT was about 110 msec longer on trials with hard choice pairs, and that relation also held for all but one subject (Figure S3 in Text S1).

### Modulation of preference by the decoy

**Figure 1:**
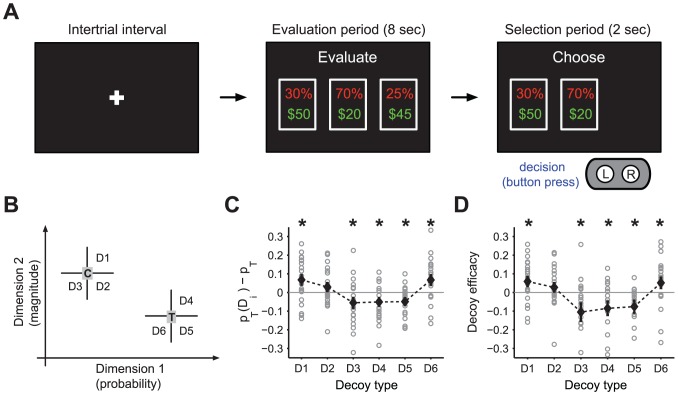
Experimental design and behavioral results. (**A**) Timeline of the experiment during the decoy task. A trial started with a fixation point, followed by the presentation of three options (monetary gambles) on the screen for 8 sec (evaluation period). These gambles were the *target* (T) and the *competitor* (C) gambles, tailored to be equally preferable, and a third gamble, the decoy (D). At the end of evaluation period, one of the three gambles was removed from the screen and subjects had only 2 sec to choose one of the two remaining gambles by pressing a button (selection period). (**B**) Positions of decoys with respect to T and C. Decoys were presented in different locations of the *attribute space*: probability (dimension 1) and magnitude (dimension 2). For data analysis, decoys were grouped into 6 locations, depending on theirs position with respect to the closest gamble to them. Decoys at D1 and D4 regions are referred to as the *asymmetrically dominant*. Decoys at D3 and D6 regions are referred to as the *asymmetrically dominated*. Finally, decoys at D2 and D5 regions are referred to as the *similar* decoys. (**C, D**) Modulation of preference for the target, and the decoy efficacy as a function of different decoys. The average of modulation for each decoy is plotted in black (error bars are the s.e.m.) and the gray symbols show the value for individual subjects. The star on a given decoy location shows that the modulation for that decoy was significantly different from zero (Wilcoxon *signed rank* test, *p<0.05*). Decoy effects were significant for all decoys except D2 decoys.

On each trial of the decoy task, three monetary gambles were displayed on the screen for an 8 sec evaluation period. At the end of this period, one of the three gambles was removed from the screen and subjects had only 2 sec to choose one of the two remaining gambles in a selection period (Figure 1A). Two of three initial gambles were the low-risk gamble (target T) and the subject-tailored high-risk gamble (competitor C). The third gamble was the decoy gamble (D) that was randomly chosen from a set of gambles with a wide range of attribute values (see Figure 1B and Methods for more details).

On two thirds of the trials (regular trials), the decoy gamble was removed after the evaluation period and the subject had to choose between T and C gambles. On the remaining one third of the trials (catch trials), either the T or C gamble disappeared. The catch trials were included to conceal the underlying structure of the task and were subsequently discarded from the analysis (since they do not provide choices between T and C). Therefore, we only analyze the regular trials to investigate how the preference between T and C gambles changed as a function of a decoy that was present at the evaluation period, but not available in the selection period.

Having a long evaluation period (8 sec) and a short selection period (2 sec) forces subjects to evaluate and “pre-choose” options by ranking them during the evaluation period; therefore, they would be prepared to make a rapid choice in the 2-sec selection period. This ensures that presentation of the decoy during the evaluation period can influence context-dependent processes of assigning values enough to have a behavioral impact during rapid selection. This “phantom decoy” design allowed us to study the effect of dominant decoys (decoys that are better than either T or C gambles) as well as dominated decoys (see below).

We found that subjects' preference between T and C was systematically influenced by the attributes of the decoys. The first indication of the decoy influence on the subsequent choice was that the majority of our subjects did not select T and C gambles equally (Figure S4 in Text S1), though they were constructed (from the estimation task data) to be equally preferable.

As in previous studies, we divided trials into 6 groups (D1 to D6) based on the position of the decoy (Figure 1B). Decoys in positions D1 and D4 are called the asymmetrically *dominant* decoys because they dominate either T or C (they are less risky and also have larger reward magnitudes), but do not dominate both. Decoys in positions D3 and D6 are asymmetrically *dominated* decoys since they are either worse than the target (D6) or the competitor (D3) on both dimensions (i.e. they are more risky and also have smaller reward magnitudes), but are only dominated by one of T and C [6], [10]. Finally, decoys in positions D2 and D5 are similar to the target and the competitor and are better on one dimension but worse on another. They are called similar decoys [28], [33].

We quantified decoy effects by computing the difference between the probability of selecting the target for a given decoy location, 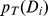, and the overall probability of choosing the target across all trials, 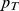 (Figure 1C). We found that the decoys influenced subjects' preference between T and C gambles (one-way ANOVA, *p<0.0001*) and the average values of 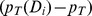 over all subjects were significantly different from zero (Wilcoxon signed rank test, *p<0.05*) except for decoys in position D2.

For statistical purposes, it was useful to scale decoy effects to account for the fact that some subjects had an overall target choice frequency, 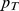, which was very different from 0.5 (despite the attempt to control this frequency using the estimation task). A scaled measured of *decoy efficacy* (see Methods) that adjusts for the target choice frequency still showed strong within-subject decoy effects (one-way ANOVA, *p<0.0005*) similar to changes in preference presented earlier (Figure 1D).

In addition, we replicated three main findings regarding decoy effects. Firstly, we observed a robust attraction effect similar to what has been shown in previous between-subject studies [6], [10]. That is, the *asymmetrically dominated decoys* D3 and D6 increased the selection of the option that dominated them: competitor C and target T, respectively (Wilcoxon signed rank test, *p<0.05*). Secondly, the *asymmetrically dominant decoy* D1 and D4 decreased the selection of the option which was dominated by those decoys: competitor C and target T, respectively (Wilcoxon signed rank test, *p<0.05*). We were able to study this effect due to our task design where the dominant decoy disappeared during the selection time. Thirdly, decoys in positions D2 and D5 decreased the selection of the option close to them (C and T, respectively); however, only the effect of decoys in position D5 was statistically significant (Wilcoxon signed rank test, *p<0.05*). These effects have been previously described as the *similarity effects* [29], indicating that decoys take more share from the option in the choice set with which they are most similar, thereby decreasing the preference for the option similar to them.

Thus, our results confirm previous between-subject findings and extend them to a within-subject design. Most preference reversals due to differences in descriptions, procedures or context are established by between-subject designs. Preference for between-subjects designs is guided by the intuition that two conditions that change a normatively irrelevant detail will be transparently equivalent if both conditions are presented in a within-subjects design; however, the normative irrelevance is cognitively inaccessible if only one condition is presented, in a between-subjects design. Establishing context-dependence in a within-subject design therefore shows its robustness. The within-subject design also adds substantial statistical power, and allows us to compute the within-subject correlation between effects for different decoys (which a between-subject design cannot do).

We also examined relationships between the overall decoy effects, as shown by a given subject and his/her risk aversion parameters from the estimation task. We found no relationship between the overall susceptibility of individual subjects to decoys (defined as the average of absolute values of decoy efficacies for each subject) and their indifference values (*r = −0.2, p = 0.38*), or between the overall susceptibility and the sensitivity to the reward magnitude (*r = −0.21, p = 0.37*).

### Dependence of decoy effects on distance and correlation between decoy effects

**Figure 2:**
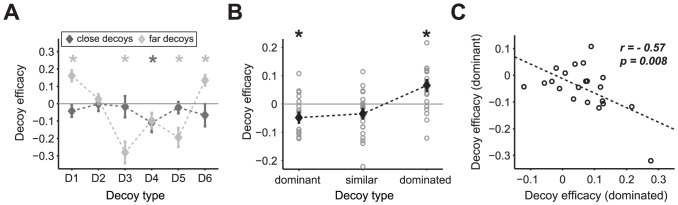
Correlation between decoy effects and dependence of decoy effects on the distance. (**A**) Dependence of the decoy efficacy on the distance of decoy from its closest option. The mean decoy efficacy for each decoy type is plotted for close (gray) and far trials (light gray), separately. The error bars are the s.e.m. and the light or dark gray star shows an effect is significantly different from zero for the corresponding location and distance (Wilcoxon signed rank test, *p<0.05*). For all subjects, decoy efficacies are larger in magnitude for far decoy trials than close decoy trials, for all decoys except D2 and D4 where they are indistinguishable. (**B**) Mean decoy efficacies for different decoy types: dominant, similar, and dominated. Each circle represents the decoy efficacy for an individual subject and the error bars are the s.e.m. The star on a given decoy type shows that the decoy efficacy is significantly different from zero (Wilcoxon signed rank test, *p<0.05*). (**C**) Anticorrelation between efficacies of dominant and dominated decoys. The dashed line shows the linear fit.

Next, we divided all regular trials into close and far trials, depending on the distance between the decoy and the gamble closest to it. Then we computed the decoy efficacy for each decoy location (Figure S5 in Text S1). For this analysis, decoy efficacies for *close* and *far* decoys were defined relative to the overall probability of selecting T only for the corresponding set of close or far decoys; therefore, this definition controlled for possible differences between the close and far sets of gambles. Close decoys had no significant effect (one-way ANOVA, *p = 0.69*), while far decoys had a very strong effect (one-way ANOVA, *p<10^−11^*) (Figure 2A). Moreover, for all decoys with significant effects over all trials (except D4), the far decoy effect was larger than the close decoy effect (two-sample t-test, *p<0.01*).

We then examined the correlation between different decoy effects within-subjects. This correlation analysis provided a tool for testing whether different types of decoy effects were generated by the same mechanisms or not. We grouped decoys at different locations into three decoy types—asymmetrically dominant decoys (D1 and D4), similar decoys (D2 and D5), and asymmetrically dominated decoys (D3 and D6). We then computed the average decoy efficacy for each of these three decoy types in terms of their effects on the preference for the gamble close to or far from them. A positive (or negative) decoy efficacy means an increase (or decrease, respectively) in the preference for the gamble close to the decoy with respect to the gamble far from it.

The different decoy types do influence the choice preference differently (one-way ANOVA, *p<0.0001*). Specifically, asymmetrically dominant decoys decreased preference for the gamble close to it (Wilcoxon signed rank test, *p<0.05*) while asymmetrically dominated decoys increased preference for the gamble close to it (Wilcoxon signed rank test, *p<0.05*) (Figure 2B). There were no significant effects for similar decoys (Wilcoxon signed rank test, *p = 0.07*). Interestingly, we found a significant negative correlation between asymmetrically dominant and asymmetrically dominated decoy efficacies (*r = −0.57, p = 0.008*) (Figure 2C).

### Behavior and predictions of the CDA model

Next we tested whether the CDA model could reproduce the decoy effects observed in our experiment. First, we briefly describe the CDA model of Tversky and Simonson [7] and we present some results and predictions of this model that are relevant to our experimental paradigm. For simplicity, we assumed options have only two attributes and that the overall subjective value of an option is a weighted sum of its values on these attributes. The latter was assumed to avoid altering the original CDA model for the case where the overall value of an option is the product of its attribute values (as for risky gambles).

In the CDA model, the context effects arise from pairwise comparison of all options in the choice set. This pairwise comparison is performed through computing quantities termed the *advantage* and *disadvantage*. More specifically, the advantage of option *T* with respect to option *C*, 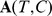, is defined as
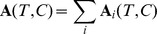
where
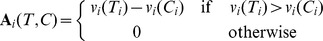
Similarly, the disadvantage of option *T* with respect to option *C*, 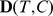 is defined as
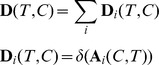
where 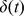 is an increasing monotonic function of 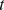 (note the change in the order of *T* and *C* in the argument of the advantage and disadvantage functions). Tversky and Simonson included loss aversion in their model, by assuming that the disadvantage looms greater than the advantage, that is 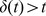 [7]. For simplicity, we assume a linear relationship, 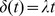 where 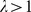.

The advantage and disadvantage are used to define the *relative advantage* of option *T* with respect to option *C*, 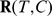


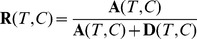
(1)Finally, the value of an option in the choice set increases proportionally to the sum of the *relative advantages* between that option and each other option in the choice set. With three options *T*, *C*, and *D*, the overall values of options including context effects are
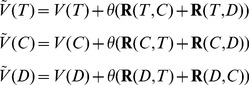
(2)where 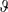 determines the strength of the context effects, and 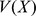 and 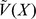 are the subjective values of option *X* before and after including the context effects. We can apply a sigmoid function to the difference in option values of T and C to obtain the choice preference between these options, before and after the decoy introduction.

**Figure 3:**
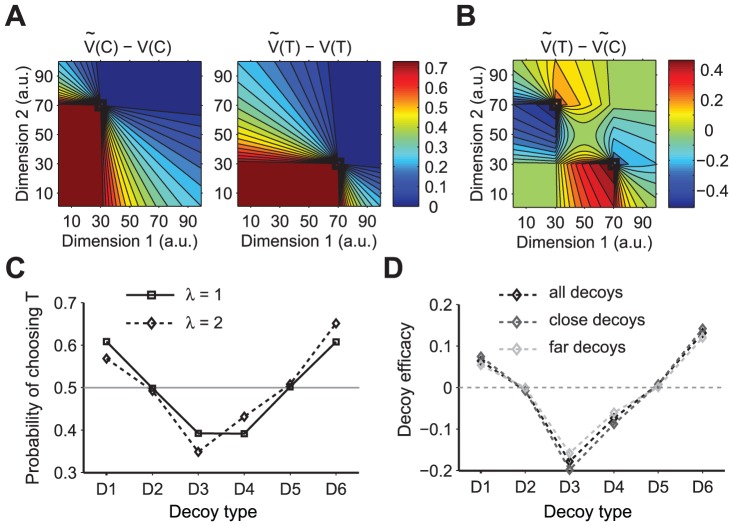
Effects of the decoy on valuation in the CDA model. (**A**) Predicted change in the overall value of the *target* (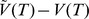) and its *competitor* (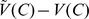) as a result of decoy introduction at different locations of the attribute space. The change in the overall value of each option and their difference is normalized by the value of these options before the inclusion of the context effects. The decoy introduction results in maximal (zero, respectively) change in the overall value of a given existing option, if both decoy attributes are smaller (larger, respectively) than the attributes of that option. (**B**) The predicted difference between the values of *target* and *competitor* (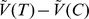) as a result of decoy introduction at different locations of the attribute space. Conventions are the same as in **A**. Introduction of the decoy results in preference reversal for many decoys while this effect is stronger for decoys that are closer to the original options than the farther decoys. Note that the effect is stronger when the decoy is dominated by the close option than when the decoy is dominant to that option (for this simulation we set 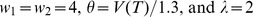). (**C**) The probability of selecting T for different decoys, as defined in Figure 1, for two realizations of the CDA model: without the inclusion of the loss aversion (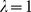) and with moderate loss aversion (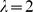). Dashed lines are to guide the eye. In both cases, CDA model predicts the attraction and asymmetrically dominant effects (i.e. reversal for D1, D3, D4, and D6) but no effects for D2 and D5 decoys. (**D**) Decoy efficacies for different decoys predicted by the CDA model with moderate loss aversion (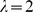) and separately for close and far decoys. Decoy efficacies are smaller in magnitude for far decoys than close decoys.

In order to illustrate the behavior of the CDA model over a wide range of decoy attributes, we calculated the change in the value of original options (i.e. the options of the choice set before the decoy was introduced) as a function of each decoy's attributes (Figure 3A). This analysis showed that the maximal change in the value of a given option happens when the decoy is dominated (both decoy attributes are smaller than the attributes of that option). Likewise, when the decoy is dominant (both decoy attributes are larger than the attribute of a given option), the change in that option value is zero, independent of the exact location of the decoy. These option value changes happen because the relative advantage is one for dominated decoys and zero for dominant decoys. Overall, decoy introduction can only add a non-negative amount to the value of original options in the choice set. This property has undesirable consequences, which we discuss later.

Next, we computed the change in the difference between the values of the original options (and the resulting change in preference between them) as a function of the decoy attributes (Figure 3B). This analysis revealed some important aspects of the CDA model. Firstly, no change in preference occurs when both decoy attributes are smaller or larger than the attributes of both of the original options. This means that in the CDA model, such decoys are irrelevant for the choice preference. Secondly, the change in preference is larger when the decoy is dominated by the close option rather than when the decoy is dominant (Figure 3B), because of loss aversion (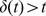). Finally, preference reversal is stronger for decoys close to the original options than for far decoys (Figure 3B).

For better comparison of the results of the CDA model with our experimental data, we calculated the average models' choice behavior for decoys at locations in the attribute space that qualitatively match our experimental design (see Methods for more details). The CDA model exhibits attraction and asymmetrically dominant decoy effects, but not similarity effects (as has been previously pointed out [26], Figure 3C). However, because both attraction and asymmetrically dominant decoy effects are driven by the same mechanism (but in an opposite direction), the values of decoy efficacies for these decoys are anti-correlated (data not shown). Moreover, as mentioned above, the decoy effects are stronger for attraction than asymmetrically dominant decoys due to the inclusion of the loss aversion concept in the CDA model (Figure 3C). There is some evidence for this prediction when we group the experimental data based on the decoy type (Figure 2B). However, fitting of our data using the CDA model yielded 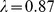, which is closer to loss-neutrality (Figure S6 in Text S1). Finally the CDA predicts that close decoys have stronger effects than far decoys (Figure 3D). This prediction of the CDA model is not supported by our experimental data (Figure 2A).

### The Range-Normalization (RN) model

Here we propose a model for context effects that can account for our experimental observations and is based on plausible limits of neuronal elements in representing sensory and cognitive stimuli. Specifically, for neural representation to be useful it should be able to distinguish between any two unequal stimuli in the set of represented stimuli. However, neural firing rates are bounded between zero and a few hundred spikes per second. That is the neural representation could be variable only in the interval between a threshold and saturation points (dynamic range); outside this interval, the stimuli are represented with the same response. Nevertheless, the response of a neuron (or a population of neurons) to a set of stimuli can still vary, depending on the relationship between the location of the threshold and saturation points and the values of all stimuli that have to be represented in the firing activity. Considering the mentioned constraints, it is therefore plausible that the response of a neuron or a population of neurons can be adjusted to a new set of stimuli that it needs to represent (widespread evidence of neural adaptation is reviewed in the Discussion). We show that this neural adjustment could explain the context-dependent preference reversal.

In this model we assumed that the overall value of a given option is represented by a neural population that receives inputs from different neural populations selective to an individual option attributes (see Method for more details). Assuming a linear response function, the overall value of an option, which is reflected in the firing activity of an option-selective population, is equal to a weighted sum of the neural responses to its attribute values
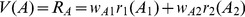
(3)where *R_A_* is the response of population selective to option *A*, *r_i_(A_i_)* is the neural response of attribute-selective population *i* to option *A*, and *w_Ai_* is the weight of connections from the attribute-selective population *i* to the option-selective population *A*.

For simplicity, we considered the case in which the neural response of attribute-selective populations is a linear function of stimulus value, *s*, when *s* is above a threshold *c_t,i_* and below a saturation point *c_s,i_*. In addition we normalized the response to the maximum response level so that the maximum response is represented with 1. Note that any difference in the maximum response of neurons encoding different attributes can be absorbed into the connection weights *w_i_'s*. Therefore, the neural representation attribute *i* can be written as
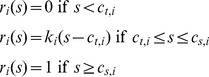
(4)and so is determined by two parameters *c_t,i_* and *c_s,i_*. In order to simplify the notation, we drop the subscript *i* in the rest of the manuscript, but it should be understood that the neural representation could be different for each attribute.

In order to express the neural response in terms of the range and configuration of represented stimuli, we define two new parameters, *f_t_* and *f_s_*, which we call the *representation factors*

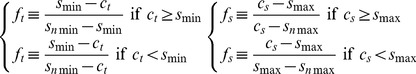
(5)where *s_min_* and *s_nmin_* are the minimum and next-to-minimum values of *s*, and *s_max_* and *s_nmax_* are the maximum and next-to-maximum values of *s*, respectively. The representation factors, *f_t_* and *f_s_*, determine the fraction of the value space around the minimum and maximum stimuli that are below or above the threshold or saturation points, respectively. This can be seen more clearly by expressing the threshold and saturation points, *c_t_* and *c_s_*, in terms of the representation factors
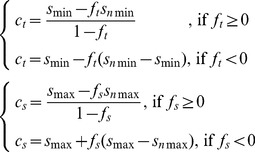
(6)Note that a positive *f_s_* implies that the neuron never reaches to its maximum possible faring rate. Therefore, the representation factors determine efficiency of a neuron (or a neural population) in representing a set of stimuli in their firing activities (see below), and so they are inherent properties of the neuron. By imposing 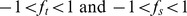, it is guaranteed that neural responses to different stimuli are distinct (except when there are only two presented stimuli, for which an additional constraint needs to be imposed: 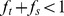).

**Figure 4:**
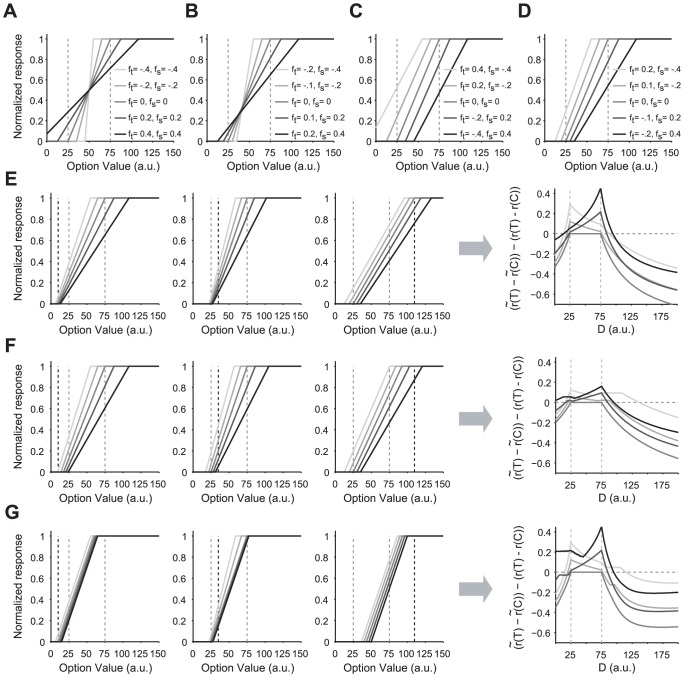
Neural representation before and after decoy introduction and its effect on the valuation process in the range-normalization model. (**A–D**) Neural responses to the original options (marked with gray vertical dotted lines at 25 and 75 a.u.) for different values of representation factors. (**E**) Neural representation after the decoy introduction in the RN model and resulting changes in the differential response to T and C. In the left three panels, the decoys are presented at 10, 35, and 110, respectively (marked with black vertical lines). The most right panel shows the change in the differential response to original options after and before decoy introduction as a function of the decoy value, D. The representation factors are the same as in **D**. (**F**) Neural representation after decoy introduction in the RN model with partial adjustment and resulting changes in the differential response to T and C. Conventions are the same as in **E**. (**G**) Neural representation after decoy introduction in the RN model with slope constraint (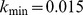) and resulting changes in the differential response to T and C. Conventions are the same as in **E**. Overall, the RN model predicts changes in the differential response to original options before and after the decoy introduction which are larger when decoys introduces a new maximum or minimum stimulus to the choice set and these effects increase with the distance, but reach to a fix value when the slope constraint is imposed.

In order to show how neural representation depends on the representation factors defined above, we plotted the neural responses for different values of representation factors in the case in which there are only two options (*C* and *T*) in the stimulus set (Figure 4A). For positive values of the representation factors threshold and saturation points are below and above the minimum and maximum stimuli, respectively. On the other hand, for negative values of representation factors, threshold and saturation points are above and below the minimum and maximum stimuli, respectively (which means extreme stimuli can be represented with the same response because they lie outside the dynamic range).

Therefore, the representation factors determine the relative position of the dynamic range of the neural response with respect to a set of represented stimuli. However, the above equations show that when a new stimulus is introduced to the stimulus set, the threshold and saturation points need to be adjusted in order for the representation factors to stay the same or adapt to the new set.

Using Eq.6 and assuming that the representation factors stay the same before and after decoy introduction (a condition which can be relaxed as shown below), we computed the adjustment of neural response and changes in the response to the original options due to decoy introduction (Figure 4B). The decoy may introduce a new minimum or maximum (or a next-to-minimum or next-to-maximum) to the stimulus set, and in all of these cases it changes the configuration of stimuli.

If there were originally two options in the set, the decoy introduction *always* changes the neural representation and therefore changes the value of the original options. More interestingly, the values of the original stimuli before and after decoy introduction depend on the relative decoy value (Figure 4B rightmost panel). This change is positive if the decoy is between the two original options or close to them, and it is negative if the decoy introduces a new minimum or maximum. Overall, the change in the differential response depends on the representation factors and decreases as the decoy becomes farther from the original options. Interestingly, we found that the ratio of the differential response after the decoy introduction to before the decoy introduction is inversely proportional to the ratio of the range of stimulus values after to before decoy introduction (see Text S1). For this reason, we call our proposed mechanism for neural adjustment the *range normalization*.

For the above simulations we assumed that adjustment to a new set of stimuli is perfect such that the neural response in terms of representation factors stay the same. However, it is possible that due to biophysical constraints, this adjustment is not fully realized (i.e. partial normalization) while neurons still represent each stimulus with different responses. To incorporate *partial range normalization*, we set the threshold and saturation points after the introduction of the new stimulus to
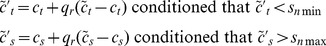
(7)where 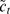 and 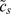 are the threshold and saturation points after the decoy introduction as described by Eq.6, and 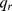 is a quantity between 0 and 1 that determines the degree of range normalization. The extra conditions assure that all stimuli are represented with different responses. If 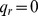, the neural response is not range normalized to the presentation of the new stimulus, and if 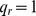, the range normalization is complete. Examples of a partial range normalization and the resulting change in the value of two original options are shown in Figure 4C (for 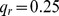). These results showed how the degree of range normalization could control the decoy effects.

A limiting factor for neural responses to distinguish between different stimuli is the ubiquitous noise in the nervous system [34]. The effects of noise on range normalization are beyond the scope of this work, however, we considered a basic consequence of noise inclusion in our range-normalization model. We assumed that in order for the neural response to be distinguishable in the presence of noise, the slope of neural response (*k*) could not be indefinitely small. Therefore, we imposed an extra constraint on the neural representation to prohibit the slope from becoming smaller than a minimum value (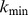). By adding this constraint to the RN model (see Methods for details), we found that the change in the differential response to original options reaches a plateau when the decoy is very far from the original options (Figure 4D). This property is psychologically plausible, however, it cannot be tested with our data since we did not use very far away decoys in our experiment.

### Behavior and predictions of the RN model

So far, we have shown how decoy introduction changes the neural response to original options based on how neurons represent a given attribute. Here we demonstrate how decoy introduction changes the preference between the original (T and C) options as observed in our experiment.

We first show how range normalization results in the attraction effect when a decoy that asymmetrically dominates T (but no C) is introduced. The difference between option values before and after decoy introduction is equal to (using Eq.3)
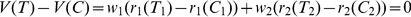
(8)

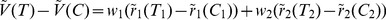
(9)where 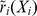 is the neural response to option *X* after the decoy introduction. By dividing the last equation by 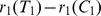 and using Eq.8 we obtain
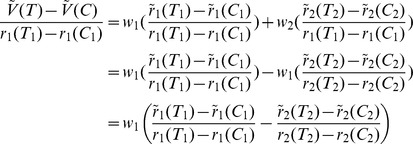
The first term in the last expression is less than one because the decoy introduces a new maximum in dimension 1, and the second term is larger than 1 as the decoy does not introduce a new minimum nor a maximum in dimension 2 (see Figure 4). Therefore, the sum of the parenthetical terms is negative so that 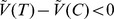, which shows that decoy introduction makes *C* preferred to *T*.

We then simulated change in preference due to decoy introduction at different locations (see Methods for details). We assumed that option attributes on a given dimension (e.g. monetary value) are represented by a neural population selective to that attribute (an attribute-selective population). The attribute-selective populations in turn project to neural populations representing the overall value of individual options (an option-selective population). The strength of these projections determines the weight of each attribute dimension on the overall value (Eq.3). Subsequently, the outputs of the option-selective populations project to a decision-making circuit, allowing the model to choose between the available options.

**Figure 5:**
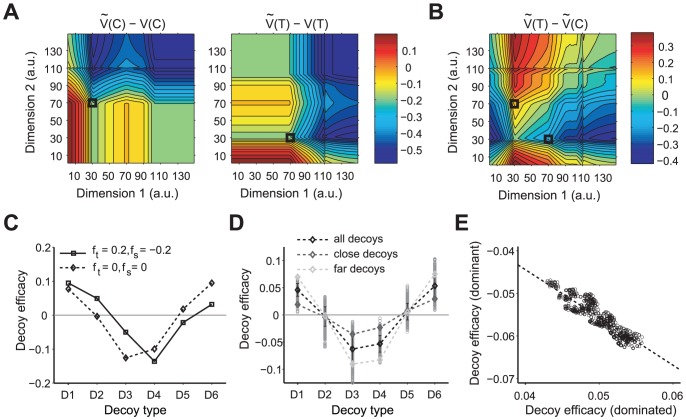
Effects of the decoy on valuation in the range-normalization model. (**A**) Predicted change in the overall value of the *target* (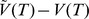) and its *competitor* (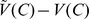) as a result of decoy introduction at different locations of the attribute space. Conventions are the same as in Figure 2A. For these simulations 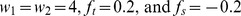 for both dimensions, and the slope constraint is imposed (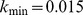). (**B**) The predicted difference between the values of *target* and *competitor* (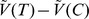) as a result of decoy introduction at different locations of the attribute space. Conventions are the same as in Figure 2B. (**C**) Decoy efficacies for different decoys for two realizations of the RN model: with representation factors equal to zero (diamonds), and with an asymmetric representation factors for the threshold and saturation (squares): 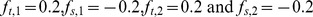. The model with asymmetric representation factors shows moderate similarity effect, in addition to the attraction and asymmetrically dominant decoy effects. (**D**) Inter-subject variability and dependence of decoy effects on the distance of the decoy from the closest option. Decoy efficacy for each instantiation of the RN model at each decoy location is shown with a gray circle and the average decoy efficacies are computed for all decoys (black diamond), close decoys (dark gray), and far decoys (light gray). Dashed lines are to guide the eye and the error bars show the standard deviation. Overall, the RN model captures the attraction and asymmetrically dominant decoy effects while it does not show significant similarity effect. Moreover, decoy efficacies are larger in magnitude for far decoys than close decoys. (**E**) Anticorrelation between dominated and dominant decoy effects in the RN model. The average decoy efficacy for dominant and dominated decoys are computed for the same set of simulations as in **D**. The dashed line shows the linear fit.

We found that the values of existing options are decreased or increased depending on the location of the decoy. These changes reach maximal values if the decoy is at a certain distance from the existing options. (Figures 5A and 5B). The fact that decoy effects do not increase indefinitely as the decoy becomes farther from the original options is due to consideration of noise in the model.

For better comparison of the behavior of the RN model with the CDA model and the experimental data, we calculated the average models' choice behavior for decoys at locations of the attribute space that qualitatively match the experimental design (the same as in Figure 5A). We found that similar to the CDA model, the RN model captures attraction and asymmetrically dominant decoy effects, but it does not capture similarity effects without including asymmetry in the representation factors of the two attributes (Figure 5C, and Figure S6 in Text S1). Interestingly, the behavior of the RN with representation factors equal to zero is qualitatively similar to the CDA model with loss-neutrality (Figure 3). In order to address between-subject variability, we simulated the model over a wide range of representation factors, and we found that overall, average behavior of many simulated subjects with this model follows the same trend as the model with zero representation factor (Figure 5D). However, in contrast to the CDA model, the decoy effects were stronger for far decoys than for close decoys. In addition, we found a significant anticorrelation between decoy effects for the attraction and asymmetrically dominant decoys (Figure 5E).

The CDA and RN models presented above, account for context effects based on very different assumptions and premises, and furthermore predict different patterns of decoy effects for far and close decoys. More importantly, different mechanisms underlying context effects in the presented models result in very different predictions regarding the influence of the choice set size on these effects, as described below.

### Biophysical plausibility and set size

Although the CDA model captures most context effects, it is unclear how computations required by this model could be implemented biologically due to two main issues. First, in order to compute the advantage and disadvantage, every pair of options in the choice set should be compared. This causes a combinatorial problem because as the choice set becomes larger the number of required comparisons grows as 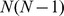, where 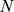 is the number of options in the choice set. Second, the CDA model asserts that the introduction of each new option results in the addition of a non-negative value to every available option in the choice set, and therefore, as the number of options in a given choice set increases the value of every option in that set increases. This implies that the value of an option not only depends on other options in a given choice set but also on the size of that set.

**Figure 6:**
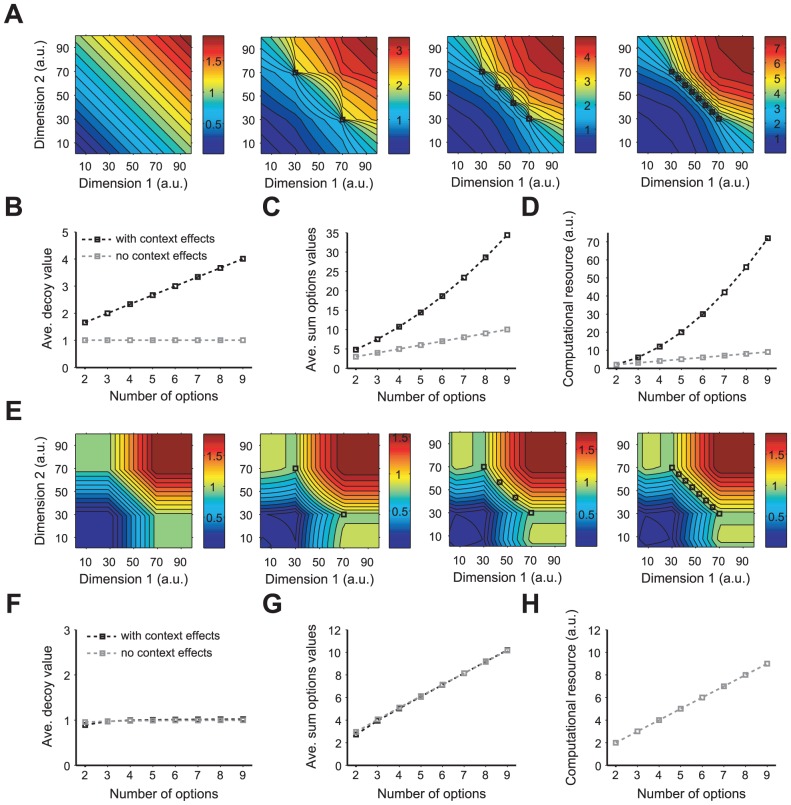
Dependence of the context effects on the size of the choice set in the CDA and RN models. Conventions are the same as in Figure 3 (**A–D**) Predictions of the CDA model. The overall value of an option in different locations of the attribute space is plotted for different choice set size: (from left to right) zero or in the absence of context effects, two, four, and eight options. The option value monotonically increases with the set size for any locations of the attribute space. (**B**) The average decoy value (over the attribute space) as a function of the choice set size in the presence and absence of the context effects. For each point we average the decoy value over all locations of the attribute space in panel **B**. (**C**) The average of the sum of option values in the choice set as a function of the choice set size. Conventions are the same as in **B**. The total options values exponentially increases with the choice set size. (**D**) Required computational resources for the network counterpart of the CDA model. Conventions are the same as in **B**. The computational resources required for context effects also exponentially grow with the choice set size. (**E–H**) Predictions of the RN model. In the RN model, the range of possible values of an option does not depend on the choice set size. (**F**) The average decoy value as a function of the choice set size. Conventions are the same as in **B**. The average decoy value does not depend on the choice set size. (**G**) The average of the sum of option values in the choice set as a function of the choice set size. The overall value of options in the choice set only increases linearly with the choice set size. Note the difference in scale in **G** and **C**. (**H**) Required computational resources for the RN model. In contrast to the CDA model, computational resources required by the RN model only increases linearly with the choice set size.

In order to illustrate the effect of the set size on the valuation in the CDA model, we computed the value of an option at different locations of the attribute space as a function of the number of equally preferable options in the choice set. We found that option value increases linearly with the number of options in the choice set, in every location of the attribute space (Figure 6A). This is a direct consequence of the fact that in the CDA model, the relative advantage always adds a non-negative value to the overall value of a given option. Therefore, the same option has a larger value when it is part of a larger choice set (Figure 6B); in addition, the overall value of the options in the choice set exponentially increases with the choice set size (Figure 6C). The former suggests that the difference between the values of two options in a given choice set should grow as the set size increases, resulting in better value discrimination in a larger choice set.

The underlying mechanisms for context effects, which rely of pairwise comparison between all options in the choice set, imply that required resources for computations of context effects should increase supra-linearly with the choice set size. To demonstrate this point, we used the network structure in the LCA model [24] to calculate the required computational resource in the CDA model or any of its equivalent neural models (see Methods for more details). We found that computational resources also increase exponentially with the choice set size (Figure 6D).

Finally, we explored the influence of the set size on the valuation in the RN model by computing changes in valuation due to decoy introduction for different number of options in the choice set (Figure 6E). We found that choice set size does not have a significant effect on valuation, and the overall value of the decoy does not change with the choice set size (Figure 6F). Moreover, the overall value of options in the choice set as well as the required computational resources increase only linearly with the choice set size (Figures 6G and 6H). These happen in our model because the computations required for context effect do not require comparison and only depend on the configuration of option values in individual dimensions. Therefore, in contrast to the CDA model, the RN model does not predict an increase in the option values as the choice set size increases. These contrasting predictions of the model can be tested in future experiments.

Table 1 summarizes the overall decoy effects predicted by the CDA and RN models, and the actual effect sizes for different decoy types. Most effects are in the predicted direction and are significant. Note that the RN model correctly predicts both the influence of distance on the decoy effects and the anti-correlation between the effects for attraction and asymmetrically dominant decoys.

**Table 1:** Predicted properties of the CDA and RN models, and associated empirical evidence.

	Property	Effect magnitude	CDA prediction	RN prediction
Effects of decoy types on choice of nearby option (decoy efficacy)				
1	Asymmetrically dominant: D1, D4, overall	−0.06*, −0.09*, −0.05*	−	−
2	Asymmetrically dominated: D3, D6, overall	0.11*, 0.05*, 0.07*	+	+
3	Similarity: D2, D5, overall	−0.03, −0.08*, 0.03	0	0†
4	Difference in magnitude of decoy efficacy of far and close decoys: D1, D4, D3, D6	0.20*, −0.01, 0.26*, 0.20*	−	+
5	Correlation of effects in rows 1 and 2	r = −0.57, p<0.008	−	−
New predictions				
6	Computational demands as a function of the choice set size	n/a	Convex	Linear
7	Choice value as a function of the choice set size	n/a	Exponential increase	Invariant

Note:

*: Denotes significant at *p<0.05*.

**†:** The RN model shows weak similarity effects but this is ignored here for simplicity. Data for rows 1–3 is from Figures 1D and 2B; row 4 is from Figure 2A; and row 5 is from Figure 2C. Results in row 6 and 7 are shown in Figure 6.

## Discussion

The prevalent influence of context on decision-making has long been considered an “anomaly” against the normative account of human choice behavior [35], [36]. The reason is that normative theories of choice typically assume that values are computed independently for each stimulus, rather than comparatively. The guiding metaphor for these normative theories of valuation and choice is a naïve theory of perception in which separate valued objects are perceived as encapsulated units and then integrated by a decision architecture. Of course, this view tends to disregard decades of evidence about how the visual system uses top-down encoding, neural adaptation and normalization, and gestalt principles in integrating multiple percepts.

In this spirit, we propose that context effects are a natural consequence of the biophysical limits of the neural processing in the brain, as shown for other aspects of perception and choice [37]–[39]. We construct a model for context effects based on plausible biophysical mechanisms that enable neurons to efficiently adjust their responses to the set of available stimuli. Both the effects of context on neural representation and the normalization to the set of stimuli have been extensively documented in auditory [40], [41] and visual domain [42]–[44], where neurons are required to represent and encode external stimuli presented in very different backgrounds. Moreover, adaptation is an efficient way for the nervous system to adjust to variable statistics of the environment to improve its local information capacity or discriminability power [45]–[50].

In our model, we explored one possible class of neural adjustments (range normalization) during valuation and choice using two main assumptions. First, neurons utilize their entire biophysical dynamic range to represent a set of stimuli. However, it is possible that neurons never reach to their maximum biophysical firing rates and instead fire at medium rates under many conditions (i.e. stimulus set). This only implies that the upper representation factor, *f_s_*, should be positive (see Eq.5) and does not qualitatively change the behavior of our model. Similarly, neurons not representing any stimulus with zero firing rate only implies positive values for the lower representation factor, *f_t_*. Second, we assume that range normalization only depends on configuration of the stimulus set and not the number of stimuli. Incorporating other parameters into response-normalization mechanisms does not contradict our proposal but it may change the resulting context effects. Here we only consider one form of range normalization to explain some of the basic effects of context on the choice preference. Future works would explore the consequences of other types of neural adjustments on the context-dependent choice behavior (see below).

Interestingly, response normalization is not unique to sensory neurons and processing, rather it seems to be a general property of cortical computations [51], [52]. A recent electrophysiological study in primates has demonstrated that some neurons in the orbitofrontal cortex (OFC) adapt their representation of the economic values to the range of values during a given session [51]. To account for this observation, Padoa-Schioppa has proposed a “range adaptation model” in which the neurons adapt their representation (by changing their sensitivity) to the range of values, while their activity does not increase with the value range. In fact, in some circumstances OFC neurons appear to encode the value of the available options in a reference-dependent fashion by representing the relative value of each option in the set [53] while in other circumstance show invariance for changes of the menu [54]. Our proposed range-normalization model is more general than the range-adaptation model and differs from this model in terms of the timescale on which adaptation or normalization takes place. That is, only in a special case where the representation factors are equal is the ratio of difference in response to original options after the decoy introduction to before the decoy introduction inversely proportional to the ratio of the range of values after to before decoy introduction (see Text S1). However, in the RN model, adjustment happens on every trial with three options. In contrast, in the range-adaptation model, the range of values on a given session controls the adaptation. It is highly possible that we would also observe such adaptation on a larger timescale (e.g. a session) if the option set changed between sessions.

Another recent study has shown that neurons in the lateral intraparietal cortex (LIP) show context-dependent effects by encoding the values of the saccade in the response field relative to the value of all other alternative saccade movements [52]. The authors used a divisive normalization model to account for their experimental findings. More specifically, the response to the value of the saccade in the receptive field is divided by the weighted response of the saccadic values of all options presented in the choice set, similarly to what has been proposed for sensory neurons [55], [56]. Therefore, due to divisive normalization, the value of each given option is globally scaled by the value of all the alternative options. In contrast, in our range-normalization model, the representation of each attribute dimension depends on the set of presented values, and not their sum (Figure 5B). Divisive normalization can account for relative value coding but does not predict any type of attraction effect because decoy introduction always suppresses the response to the target and the competitors without any change in the ranking of the options. However, it is possible that our proposed range normalization and the divisive normalization mechanisms play roles during different stages of decision process. Range normalization operates at the early stage of the decision process when cortical neurons have to represent individual features of each option; while divisive normalization operates at final stages (e.g. in LIP) when overall value associated with different actions need to be represented to control the selection processes (e.g. saccades).

A number of psychological models have used the attribute comparison as the basic mechanism to account for attraction and other decoy effects. The CDA model presented here was chosen as an example of such models because it accounts for the attraction and asymmetrically dominant decoy effects and provides testable predictions due to its simple, yet clear mathematical formulation. However, the CDA model or any other model that relies on attribute comparison, suffers from a few important issues. Firstly, such models predict that the values of all options increase (or at least the best and worst option) as the choice set increases, which implies that when presented as part of larger choice set options can be differentiated easier than when they are presented in a smaller set. This prediction is in contrast with experimental evidence showing that discriminability between items decreases with the increase of the data set [57], and that neural representation of option values decrease as the number of alternatives increase [52]. Secondly, in such models, resources required for computation of context effects exponentially increases with the choice set. The CDA model also predicts that decoy effects are larger for closer decoys. This is somehow counterintuitive as it predicts maximal decoy effects for very similar but dominated decoys - while these decoys should have little or no effect on the preference for the close dominant option, as it might be hardly distinguishable.

Recently, more sophisticated connectionist models have been proposed to capture attraction and other context effects such as the compromise and similarity effects. Two of such connectionist models are the decision-field theory (DFT) [26] and leaky competing accumulator (LCA) models [24]. While in both models attention determines which attribute to be compared at the time, these models rely on different mechanisms to account for attraction effect. The DFT model relies on bi-directional distant-dependent inhibition while the LCA model depends on the loss aversion. However, because both the DFT and LCA models require attribute comparison at some stages of processing (similar to the CDA model), they both suffer from the combinatorial problem as the CDA model. In contrast, our model that relies on range normalization of neural responses, which is adjusted only once regardless of the number of options, does not suffer from this issue.

There are other psychological models of context effects that do not rely on attribute comparison as the basic mechanism. Most of these models are based on heuristics and are not mathematically well formulated. These include but are not limited to the so-called weight-change, value-shift, and value-added models [21]. The weight-shift model assumes that adding a new alternative changes the relative weights of different attributes; it reduces the weight of a given attribute if the range on that attribute is extended and increases the weight if the number of different attribute values is increased. The value-shift model on the other hand, assumes that decoy changes the subjective evaluation of the attribute values, mainly based on the relative position of decoy with respect to the rest of options (as in range-frequency theory [58]). Finally the value-added model assumes that decoy introduction adds values to original options, which depend on the relational properties of the decoy and each target. Our range-normalization model shares some similarities with the value-shift model in a sense that it assumes that the decoy value on a given attribute changes the value representation in that attribute independently of the other attributes. However, for a limited case where representation factors are equal, the effective weight of a given dimension is inversely proportional to the range of values on that dimension (but there is no explicit relationship to the frequency effects in weight-shift model). Despite this similarity, our model relies on very different assumptions to explain the decoy effects and generates a number of novel predictions, while it is difficult to generalize the previous models because of their lack of mathematical formalization.

Still another set of models, from economics and marketing [59]–[61], assume that consumers are not sure what they prefer, but those consumers *infer* reasonable preferences from what options are available (as if mere option availability is advice). Decoys have an influence because they shape the consumer's idea of what might be a good choice. Comparison of these models with the CDA, RN and others is an interesting area for future research.

Context is a powerful modulator of how underlying preferences are constructed and choices are made, as documented by many behavioral experiments and field studies [35], [62]. At the theoretical level, however, most of the attempts to account for context effects have neglected the computational constraints faced by the brain in order to compare choice options characterized by several different attributes. In this paper we show that considering plausible biophysical constraints of the nervous system can indeed account for a few important aspects of context effects. The range-normalization model we proposed here has a reduced computational cost relative to competing models and at the same time produces accurate empirical predictions. More importantly, it enables us to connect plausible biophysical constraints of neural representation to the biases in the human choice behavior.

## Methods

### Ethics statement

All participants gave informed consent to participate according to a protocol approved by the California Institute of Technology Institutional Review Board.

### Experimental paradigm and subjects

The experiment consisted of two parts in which subjects selected between different monetary gambles. In the first part (estimation task), the subject selected between two gambles with different reward probabilities and magnitudes. We used subject's choice in this task to estimate his/her attitude toward risk and to tailor equally preferred target (T) and competitor (C) gambles. In the second part of the experiment (decoy task), we assessed the preference between the target and competitor gambles in the presence of a third gamble. The subjects were told to consider every trial as equally important because at the end of the experiment, only one trial would be randomly extracted and the selected gamble on that trial would be played for real. To further encourage subjects to pay attention to every trial, we deducted $1 from the final compensation for each missed response.

In total, 22 healthy Caltech male students (22±4 years old) took part in the study. One subject was excluded from the data analysis since he showed an erratic pattern of gamble selection during the estimation task. This was reflected in a poor fit of his choice behavior - his sensitivity to reward magnitude, 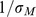, was 7 times smaller than the mean of the group (see Figure S2 in Text S1 for the distribution) - which prevented a reliable estimation of his indifference point.

### Estimation task

In the estimation task, we assessed individual subjects' risk attitude using selection between two monetary gambles. The assessment procedure was an adaptation of the widely used method for estimating the indifference point which was originally developed by Holt and Laury [63]. Every subject completed four equivalent sessions, each of which consisted of 40 trials. On each trial, the subject had 4 seconds to evaluate two gambles while the instruction message “Evaluate” was on the screen. After this interval, the instruction message was changed to “Choose” and the subject had 2 seconds to indicate their choice using a keyboard. Each gamble was defined by two parameters (*p, M*), probability *p* of winning a monetary reward of magnitude *M*, that were presented on the screen with different colors. One gamble was characterized by a small reward magnitude but a large reward probability (low-risk or the *target* gamble). The other gamble had a large reward magnitude but a small reward probability (high-risk or the *competitor* gamble). We fixed the magnitude and probability of the low-risk gamble (*p = 0.7, M = $20±2*) while we varied the magnitude of the high-risk gamble between $30 and $80 (*p = 0.3, M = $30–$80*).

### Decoy task

In the second part of the experiment, we tested how presence of different decoy gambles influences the preference between the low-risk and high-risk gambles. The low-risk gamble (T) was set to have a magnitude *M* of $20±2 and a probability *p* of 0.7±0.05. The high-risk gamble (C) was set to have a probability *p = 0.3±0.05* while its magnitude was tailored individually using the indifference point from the estimation task, in order to have the subjects indifferent between T and C. Finally, decoy gambles (D) were designed to have a wide range of magnitude and probability values (Figure 1). Specifically, we varied probability values of the decoy between 0.15 and 0.85, while we varied its reward magnitudes by 30% of the reward magnitude of the gamble closest to the decoy.

The task sequence was as follows. Three gambles (T, C and D) were presented on the screen for 8 seconds (evaluation period) while the “Evaluate” message was on the screen. The subjects were told to evaluate the three gambles during this period. Once the evaluation time was over, the message “Evaluate” was changed to “Choose” and simultaneously, one of the three gambles was randomly removed from the screen. The subjects then had 2 seconds to choose between the two remaining gambles by pressing a keypad (selection period). The decoy task was conducted in the MRI scanner (Siemens Trio); however, the fMRI data are neither analyzed nor presented here as they are beyond the scope of this paper. The main reason for not including the fMRI data here was that none of the models presented in this paper generates predictions that could be tested using BOLD-level signals.

On one third of the trials (catch trials), either C or T gambles disappeared. These trials were included to avoid the subject from predicting which gamble would disappear after the evaluation period, and were subsequently excluded from the analysis. On the remaining two thirds of the trials (regular trials), the decoy gamble disappeared allowing us to study how the presence of this option in the choice set influence the preference between C and T. Using this design (i.e. phantom decoy design), we were able to examine the effects of decoys that were preferred over C or T gambles. Finally, we used a short choice period (2 seconds) to avoid subjects from reevaluating the two remaining gambles. In fact, the only way to perform this task efficiently was to rank the 3 gambles during the evaluation period and to use this ranking at the choice period. Debriefing after the study confirmed that a large majority of the subjects used this “ranking strategy” which was also reflected in the dependence of the RT on the decoy (Figure S7 in Text S1).

### Range-normalization model

The range-normalization model consists of three layers of neural populations: the attribute-selective, option-selective, and decision-making populations. The attribute-selective layer consists of two neural populations that represent the two attributes of the options. The attribute-selective populations project to the option-selective layer that consists of neural populations each of which represents the subjective value of an option in the choice set. The subjective values of options are determined by the weight of connections from the attribute-selective layer to the option-selective layer (Eq.3). Finally, the outputs of option-selective populations project the corresponding populations in the decision-making layer. The decision-making network is similar to what has been previously used to simulate different reward-dependent choice behaviors [37], [64].

Here we were only interested in the outcome of decision-making processes, therefore, we did not simulate the decision-making network on every trial. Instead, we used a sigmoid function, which has shown to describe the choice behavior of the decision-making network very well [37], [64], in order to compute the choice probability for a given set of inputs to the decision network. More specifically, the probability of selecting T, 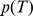, is equal to
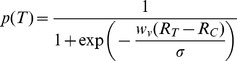
(10)where *R_T_* and *R_C_* are the responses of option-selective populations for target and competitor (Eq.3), 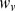 is the strength of connections from option-selective to decision-making populations, and 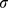 is a model parameters which is determined by the architecture of the decision-making network and the overall strength of its inputs [37], [64].

In order to obtain the neural response of attribute-selective populations to a given stimulus set, we used Eq.6 to calculate the threshold and saturation points. The threshold and saturation points uniquely define the neural response through Eq.4. To calculate the neural response after the decoy introduction, we first identified the minimum and maximum, and next to minimum and maximum stimuli in the stimulus set, and then we used Eq.6 to compute the threshold and saturation points.

For simulations presented in Figure 4C, we used Eq.7 to calculate partially adjusted threshold and saturation points. For simulations presented in Figure 4D, an additional constraint for the slope of neural response was imposed as follows. For a given decoy location, we calculated the threshold and saturation points from which the slope could be determined. If the slope was below the minimum value (0.015 in simulations presented in this paper), in a stepwise fashion we increased and decreased the values of threshold and saturations points, respectively, until the slope value became larger than the minimum slope value. In order to simulate decoy effects in the two-dimensional attribute space, the same procedure was applied on each attribute dimension independently. For simulations presented in Figure 5D and Figure 5E, the representation factors are selected from any combinations of 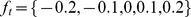
and 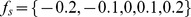 for each attribute dimension.

Finally, to calculate the required computational resource in our model, we assumed that an addition of each option to the choice set requires the engagement of one neural population to represent the subjective value of the new option, which requires an additional option-selective population. In contrast, in the network implantation of the CDA model, such as the LCA model, an addition of each option requires the engagement of a few neural populations that are required for comparison between each attribute of the new option and the existing options. As a result, required computational resources in CDA model increases with the number of options in the choice set, 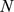, as 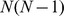. All simulations were performed using custom-made codes in MATLAB.

### Data analysis

For the statistical tests presented in the paper, we have provided the conventional significant values in addition to the applied test. In order to quantify the decoy effects, we used the overall preference for the target gamble and the preference for the target gamble for a given decoy to define the *decoy efficacy*, 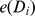


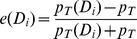
Based on this definition, the decoy efficacy is bound between −1 and 1. Note that using preference for C to define the decoy efficacy gives similar results to what presented here.

## Supporting Information

Text S1 A PDF file containing additional analysis of the CDA and RN models, and the supplementary figures.
(PDF)
