# Supplementary material for: A Range-Normalization Model of Context-Dependent Choice: A New Model and Evidence
Source: PLoS Comput Biol. 2012 Jul 19;8(7):e1002607. doi: 10.1371/journal.pcbi.1002607 (PMC3400579; doi:10.1371/journal.pcbi.1002607)
Supplement: Text S1 — A PDF file containing additional analysis of the CDA and RN models, and the supplementary figures. (PDF) [file pcbi.1002607.s001.pdf]

Supporting Information

Supplementary Note 1:

The CDA model account of the attraction effect

Here we describe how the context-dependent advantage (CDA) model accounts for the attraction effect. We assume that originally there are two equally preferable options in the choice set, called target (T) and competitor (C), and we examine how the introduction of a third option, called the decoy (D), changes the preference between the original options. In addition, each option has only two attributes and the overall value of an option is a weighted sum of its values on these attributes. Note that both the CDA and the range-normalization model can be generalized easily to the case where each option has more than two attributes and the value function is a monotonic function of attribute values. However, only the range-normalization model can be generalized for the case where the overall value of an option is the product of its values on different attributes.

Based on the above assumptions, the overall subjective value of option *T, V(T),* can be written as

$$V(T) = \sum_i v_i(T_i) = v_1(T_1) + v_2(T_2) = w_1T_1 + w_2T_2 \quad \text{(Eq.S1)}$$

where T_i is the option value on attribute *i* and weight w_i quantifies the contribution of this attribute to the overall value function. Moreover, because the original options *T* and *C* have equal overall subjective value we have

$$V(T) = V(C) \Rightarrow w_1(T_1 - C_1) = w_2(C_2 - T_2)$$

where $T_1 > C_1$, $T_2 < C_2$.

Now consider the introduction of an asymmetrically dominant option to the choice set. That is, the third option (decoy) has larger values than option *T* in both attributes, but only one of its attribute values is larger than that of option *C*

$$D_1 > T_1, D_2 > T_2 \quad \text{and} \quad D_1 > C_1, D_2 < C_2$$

Using Eq.1 and Eq.S1, for the case of the linear utility function presented here the *relative advantage* of *T* with respect to *C* is equal to

$$\mathbf{R}(T,C) = \frac{v_1(T_1) - v_1(C_1)}{v_1(T_1) - v_1(C_1) + \lambda(v_2(C_2) - v_2(T_2))}$$
$$= \frac{w_1(T_1 - C_1)}{w_1(T_1 - C_1) + \lambda(w_2(C_2 - T_2))}$$

However, $\mathbf{R}(T,D)$ is equal to zero because D dominates T in both attributes.
Similarly, the *relative advantage* of option C relative to option T and D is equal to

$$\mathbf{R}(C,T) = \frac{w_2(C_2 - T_2)}{w_2(C_2 - T_2) + \lambda(w_1(T_1 - C_1))} = \mathbf{R}(T,C)$$

$$\mathbf{R}(C,D) = \frac{w_2(C_2 - D_2)}{w_2(C_2 - D_2) + \lambda(w_1(C_1 - D_1))} > 0$$

Note that $\mathbf{R}(C,T) = \mathbf{R}(T,C)$ because $w_1(T_1 - C_1) = w_2(C_2 - T_2)$. Consequently, updated values of the original options after the decoy introduction are equal to

$$\tilde{V}(T) = V(T)$$
$$\tilde{V}(C) = V(C) + \theta\mathbf{R}(C,D)$$

where $V(T)$ and $\tilde{V}(T)$ are the values of option T before and after the decoy introduction, respectively. The last equation shows that in the presence of the decoy, option C has a larger value than option T, and so it is more preferable relative to option T. Therefore, this model accounts for the preference reversal due to introduction of an asymmetrically dominant decoy (also called attraction effect) by an extra relative advantage of C with respect to this decoy.

Supplementary Note 2:

Range-normalization model with equal representation factors

In this section, we consider a special case of our range-normalization model in which the representation factors are equal and we show that in such case the outcome normalization resembles the "range-adaptation model" of Padoa-Schioppa [1], but only on a trial-by-trial basis and not on a session-by-session basis as in the latter model. More specifically, we show that for an original option set that consists of two options when the representation factors are equal, the ratio of the difference in response to the original options after the decoy is introduced to before the decoy is introduced is inversely

proportional to the ratio of the range of option values after the decoy introduction to before it.

First let us consider the case where both f_t and f_s are smaller than or equal to zero. In this case the difference between the responses to two original options is equal to one and the slope of the neural response is equal to

$$k = 1/(c_s - c_t) = 1/(\Delta_s + \Delta_s(f_t + f_s))$$

where $\Delta_s \equiv s_{max} - s_{\min}$ is the range of stimulus values in the original set. If the new option (decoy) falls between the original options, then the difference between the responses to original options remains the same, $\tilde{r}(T) - \tilde{r}(C) = 1$, as the original options will still be below and above the threshold and saturation points. If the decoy introduces a new maximum to the choice set, then the threshold, c_t, stays the same (therefore $r(C)=0$), while the saturation point, c_s, increases. Therefore, the difference between the response to the original options, which is proportional to the slope of the neural response after the adjustment, is equal to (using Eq.9)

$$\begin{aligned}
&\tilde{r}(T) - \tilde{r}(C) = \tilde{k}(s_{\max} - \tilde{c}_t) - 0 = (s_{\max} - c_t)/(\tilde{c}_s - \tilde{c}_t) \\
&= (s_{\max} - s_{\min} + f_t(s_{\max} - s_{\min}))/(\tilde{s}_{\max} + f_s(\tilde{s}_{\max} - s_{\max}) - (s_{\min} - f_t(s_{\max} - s_{\min}))) \\
&= \Delta_s(1 + f_t)/(\tilde{\Delta}_s(1 + f_s) + \Delta_s(f_t - f_s))
\end{aligned} \qquad \text{(Eq.S2)}$$

where $\tilde{s}_{max}$ and $\tilde{\Delta}_s$ are the new maximum and the new range of stimulus values after the decoy introduction, respectively, and $\tilde{c}_t$ and $\tilde{c}_s$ are the threshold and saturation points after the decoy introduction. Similarly, if the decoy introduces a new minimum, c_s and $r(T)$ stay the same, c_t decreases, and the value of $r(C)$ increases from zero. Therefore, the difference between the responses to the original options is equal to

$$\begin{aligned}
&\tilde{r}(T) - \tilde{r}(C) = 1 - \tilde{k}(s_{\min} - \tilde{c}_t) = 1 - (s_{\min} - \tilde{c}_t)/(\tilde{c}_s - \tilde{c}_t) \\
&= 1 - (s_{\min} - \tilde{s}_{\min} + f_t(s_{\min} - \tilde{s}_{\min}))/(s_{\max} + f_s(s_{\max} - \tilde{s}_{\min}) - (\tilde{s}_{\min} - f_t(s_{\min} - \tilde{s}_{\min}))) \\
&= 1 - ((\tilde{\Delta}_s - \Delta_s)(1 + f_t))/(\tilde{\Delta}_s(1 + f_t) + \Delta_s(f_s - f_t)) \\
&= \Delta_s(1 + f_s)/(\tilde{\Delta}_s(1 + f_t) + \Delta_s(f_s - f_t))
\end{aligned} \qquad \text{(Eq.S3)}$$

Using the Eq.S2 and Eq.S3 we can see that in both cases when the representation factors are equal, $f_t = f_s$, the ratio of the difference between the responses to the original options after to the difference in responses before the decoy introduction is equal to

$$\frac{\tilde{r}(T)-\tilde{r}(C)}{r(T)-r(C)}=\frac{\Delta_s}{\tilde{\Delta}_s} \quad \text{if} \quad f_t = f_s \le 0 \qquad \text{(Eq.S4)}$$

One can perform similar calculation for the case in which the representation factors are equal and positive. We find that if the decoy is a new minimum or maximum then

$$\frac{\tilde{r}(T)-\tilde{r}(C)}{r(T)-r(C)}=\frac{\Delta_s}{\tilde{\Delta}_s}(1+f_t) \quad \text{if} \quad f_t = f_s > 0 \qquad \text{(Eq.S5)}$$

But even if the decoy does not introduce new optima, the ratio of the difference in responses after the decoy introduction to the difference before the decoy introduction is equal to $(1+f_t)$. That is, the presence of the decoy can change the preference between the two original options even if its value is between these options.

These results show that if the representation factors are equal, the ratio of the difference in response to the original options after the decoy is introduced to before the decoy is introduced is inversely proportional to the ratio of the range of option values after the decoy introduction to before it. In any case, the change in valuation due to presentation of a new option depends on the configuration of options in the choice set.

References

1. Padoa-Schioppa C (2009). Range-adapting representation of economic value in the orbitofrontal cortex. *J Neurosc* (29): 14004–14.

Supplementary Figures

Figures S1-S5 and Figure S7 provide additional analyses of the experimental data. Figure S6 provides the fitting of the CDA and range-normalization models to the main experimental data.

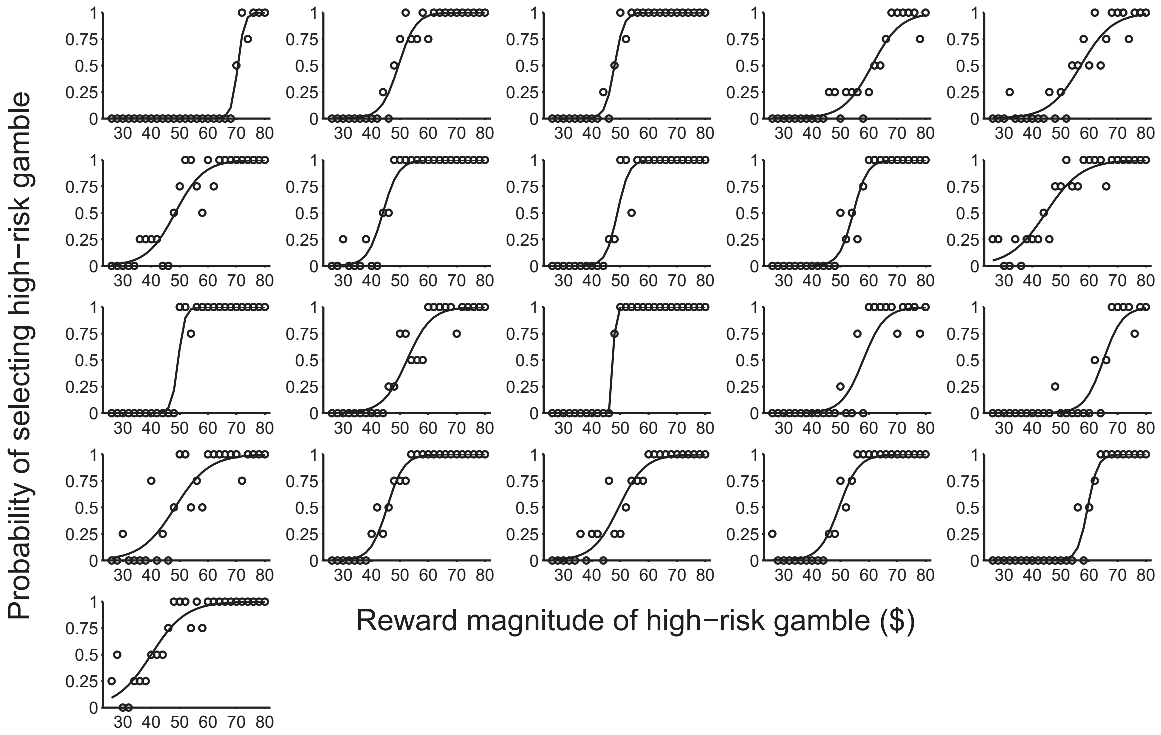

Figure S1: **The choice behavior of subjects during the estimation task.**

Plotted is the probability of choosing the high-risk gamble as a function of its reward magnitude for individual subjects (the probability of this gamble was fixed at p=0.3). Note that the magnitude and probability of the low-risk gamble were fixed at M=$20 and p=0.7, respectively. As the magnitude of the high-risk gamble was increased, it was preferred more often over the low-risk gamble. The solid curve on each panel shows the result of the logistic fit for individual subjects from which two quantities are extracted: the indifference value (the magnitude of a high-risk gamble for which the two gambles are selected equally), and the inverse of sensitivity of the each subject to the reward magnitudes (see Figure S2).

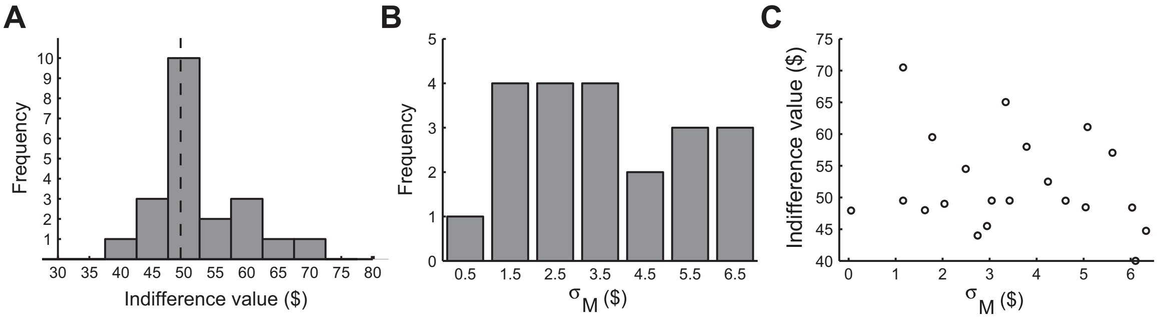

Figure S2: **The choice parameters during the estimation task.**

(**A**) The distribution of the indifference point (i.e. the magnitude for which the high-risk gamble is selected with equal probability as the low-risk gamble) for all subjects. The dashed line shows the median of the distribution. (**B)** The distribution of the inverse of sensitivity to the reward magnitudes, σ_M . (**C**). The indifference points as a function of σ_M . There was no correlation between the two parameters.

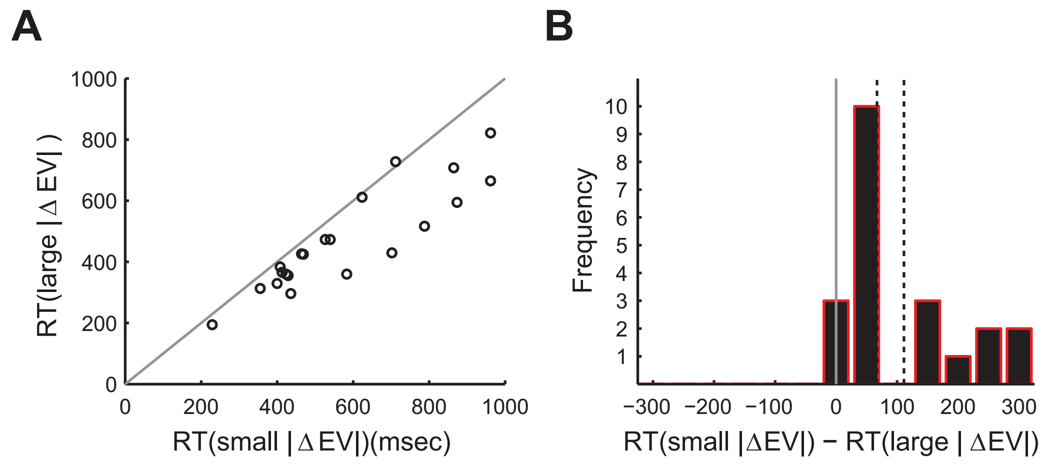

Figure S3: **Dependence of the response times (RTs) on the difference between the relative expected value (EV) of the two gambles during the estimation task.**

(**A**) Each circle shows the mean RTs for trials on which the difference between expected values of gambles were large (easy trials), ΔEV larger than two times σ_{EV}, versus the RTs for trials on which the difference between expected values of gambles were small (hard trials), ΔEV smaller than or equal to two times σ_{EV}, for each subject. All subjects (except one) showed slower RTs on hard trials where the subjective values of two gambles were close to each other. The gray line is the diagonal line. (**B**) Histogram of the difference between the RTs on two types of trials described above. The dashed lines show the median and mean of the distribution at ~67 msec and ~111 msec, respectively.

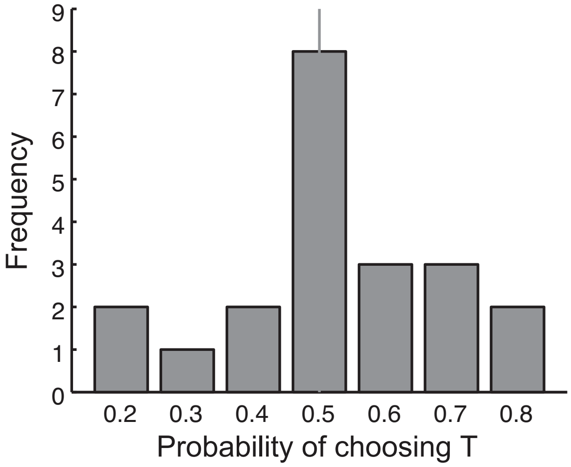

Figure S4: **Probability of choosing the target gamble during the decoy task.**

Plotted is the histogram of the choice probability (i.e. the probability of choosing the target gamble) of all subjects during the decoy task. While the target and competitor gambles were designed to be equally preferable (i.e. p_T=0.5), a number of subjects selected these gambles unequally. This pattern of preference indicated that decoys had a strong effect on choice preference.

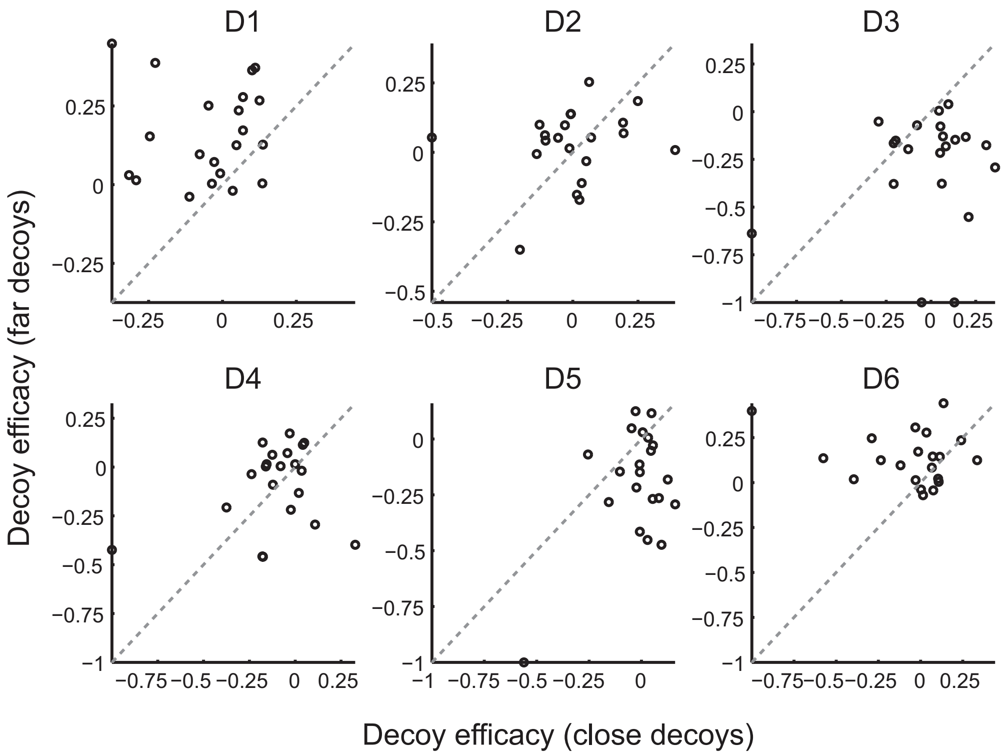

Figure S5: **The effect of distance on the decoy efficacy.**

Each panel shows the decoy efficacies for close and far decoy trials for a given decoy location (D1 to D6), for each individual subject. For all decoy locations, except D2 and D4, the decoy efficacies for far decoys were larger in magnitude than the decoy efficacies for close decoys. Dashed lines are the diagonal lines in each panel.

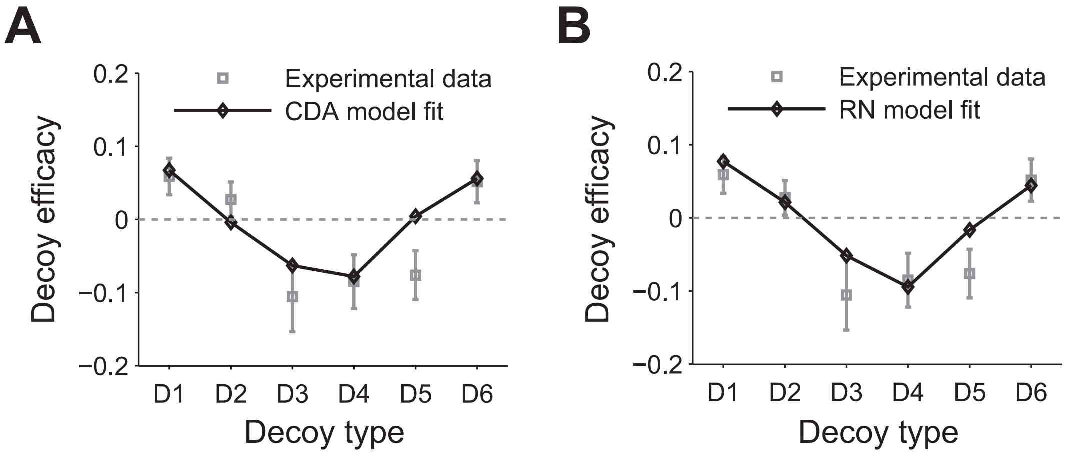

Figure S6: **Fit of the experimental data with the CDA and RN models.**

(**A**) Fit of the average behavior of all subjects with the CDA model with two parameters, λ and θ. The best fit yields $\lambda = 0.87$ and $\theta = 0.27$ with $\chi^2 = 0.40$. (**B**) Fit of the average behavior of all subjects with the RN model with two parameters, f_t and f_s (equal for both attributes). The best fit yields $f_t = 0.37$ and $f_s = 0.31$ with $\chi^2 = 0.25$. For both fits we used $\sigma = 0.40$.

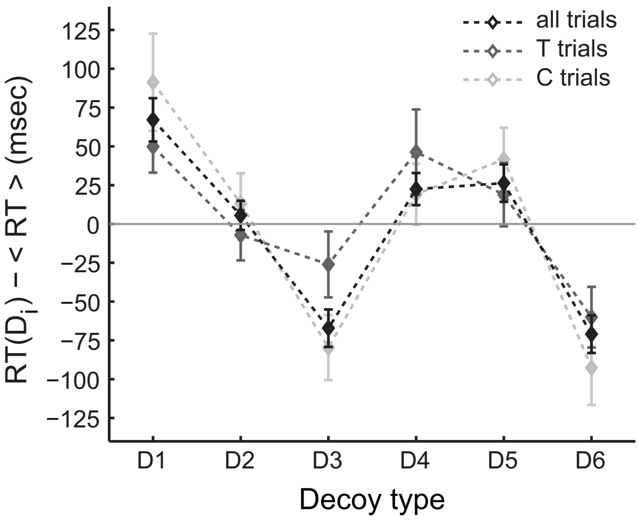

Figure S7: **The effect of decoy on the response time during the decoy task.**

The relative RT (average RT for a given decoy location minus the average RT over all trials) is plotted for different decoy locations, and separately for trials on which T or C gambles were selected. The relative RT was significantly different from zero (Wilcoxon signed rank test, $p<0.05$) for all decoys except D2 (the location for which the decoy effect was not significant). For dominant decoys the RT was positive, which shows that subjects were slower when their most preferred gamble was removed before the selection. For dominated decoys the relative RT was negative, which shows that subjects were faster when their least preferred gamble was removed before selection. We found no difference in relative RT for trials on which T or C gambles were selected. Overall, these results suggest that subject used “ranking” to perform the task.
